# Supplementary material for: Circulating Tumor DNA Early Kinetics Predict Response of Metastatic Melanoma to Anti-PD1 Immunotherapy: Validation Study
Source: Cancers (Basel). 2021 Apr 11;13(8):1826. doi: 10.3390/cancers13081826 (PMC8069589; doi:10.3390/cancers13081826)
Supplement: Supplementary file 1 [file cancers-13-01826-s001.pdf]

cancers

Article

Circulating Tumor DNA Early Kinetics Predict Response of Metastatic Melanoma to Anti-PD1 Immunotherapy: Validation Study

Guillaume Herbreteau [1,2], Audrey Vallée [1,2], Anne-Chantal Knol [2], Sandrine Théoleyre [1,2], Gaëlle Quéreux [2,3,4], Emilie Varey [2,4], Amir Khammari [2,3,4], Brigitte Dréno [2,3,4] and Marc G. Denis [1,2,*]

1 Laboratoire de Biochimie et Plateforme de Génétique Moléculaire des Cancers, CHU Nantes, 44093 Nantes, France; guillaume.herbreteau@chu-nantes.fr (G.H.); audrey.vallee@chu-nantes.fr (A.V.); sandrine.charpentier@chu-nantes.fr (S.T.)
2 Centre de Recherche en Cancérologie et Immunologie, CRCINA, INSERM U1232, 44007 Nantes, France; anne-chantal.knol@univ-nantes.fr (A.-C.K.); gaelle.quereux@chu-nantes.fr (G.Q.); emilie.varey@chu-nantes.fr (E.V.); amir.khammari@chu-nantes.fr (A.K.); brigitte.dreno@atlanmed.fr (B.D.)
3 Service de Dermatologie, CHU Nantes, 44000 Nantes, France
4 Centre d'Investigation Clinique INSERM CIC1413, CHU Nantes, 44021 Nantes, France
* Correspondence: marc.denis@chu-nantes.fr; Tel.: +33-240-08-40-02; Fax: +33-240-08-39-91

Citation: Herbreteau, G.; Vallée, A.; Knol, A.-C.; Théoleyre, S.; Quéreux, G.; Varey, E.; Khammari, A.; Dréno, B.; Denis, M.G. Circulating Tumor DNA Early Kinetics Predict Response of Metastatic Melanoma to Anti-PD1 Immunotherapy: Validation Study. *Cancers* **2021**, *13*, 1826. https://doi.org/10.3390/cancers13081826

Academic Editor: Giulia Siravegna

Received: 6 March 2021
Accepted: 9 April 2021
Published: 11 April 2021

Publisher's Note: MDPI stays neutral with regard to jurisdictional claims in published maps and institutional affiliations.

Copyright: © 2021 by the authors. Licensee MDPI, Basel, Switzerland. This article is an open access article distributed under the terms and conditions of the Creative Commons Attribution (CC BY) license (http://creativecommons.org/licenses/by/4.0/).

Table S1. Characteristics of first-line patients.

	Total	Undetectable Baseline ctDNA	Detectable Baseline ctDNA	*p*
n ($n_{derivation}$ + $n_{validation}$)	58 (26 + 32)	33 (14 + 19)	25 (12 + 13)	-
Age<br>m (Q1—Q3)	65.5<br>(59.5—75.7)	66.6<br>(60.4—79)	64.1<br>(59—72)	0.520
Tumor thickness<br>m (Q1—Q3)	3.6<br>(1.6—5)	3.3<br>(1.7—4.9)	4<br>(1.4—5.8)	0.862
Number of metastases<br>m (Q1—Q3)	2.8<br>(2.0—3.8)	2.1<br>(2—2)	3.8<br>(2—5)	<0.0001
Baseline LDH<br>IU/L; m (Q1—Q3)	381.0<br>(180.1—414.5)	204.3<br>(174—227.2)	539.9<br>(203—516.5)	0.003

		Total	Undetectable Baseline ctDNA	Detectable Baseline ctDNA	*p*
Gender	M	22	7 (32%)	15 (68%)	0.006
	F	36	26 (72%)	10 (28%)	
Stage	III	24	19 (79%)	5 (21%)	0.007
	IV	34	14 (41%)	20 (59%)	
Ulceration	Yes	21	11 (52%)	10 (48%)	0.848
	No	21	13 (62%)	8 (38%)	
Presence of lymph node metastasis	Yes	41	19 (46%)	22 (54%)	0.019
	No	17	14 (82%)	3 (18%)	
Presence of cutaneous metastasis	Yes	33	19 (58%)	14 (42%)	1.000
	No	25	14 (56%)	11 (44%)	
Presence of pulmonary metastasis	Yes	14	5 (36%)	9 (64%)	0.120
	No	44	28 (64%)	16 (36%)	
Presence of cerebral metastasis	Yes	6	3 (50%)	3 (50%)	1.000
	No	52	30 (58%)	22 (42%)	
Presence of abdominal metastasis	Yes	15	3 (20%)	12 (80%)	0.002
	No	43	30 (70%)	13 (30%)	
Presence of bone metastasis	Yes	15	2 (13%)	13 (87%)	0.0002
	No	43	31 (72%)	12 (28%)	
Mutated gene	NRAS	51	28 (55%)	23 (45%)	0.687
	BRAF	7	5 (71%)	2 (29%)	
Baseline LDH	≥426 IU/L (2x ULN)	9	0	9 (100%)	0.001
	<426 IU/L (2x ULN)	29	18 (62%)	11 (38%)	
	Undetermined	20	15 (75%)	5 (25%)	
Treatment	Nivolumab monotherapy	51	30 (59%)	21 (41%)	0.450
	Nivolumab + Ipilimumab	7	3 (43%)	4 (57%)	

ULN: upper limit of normal

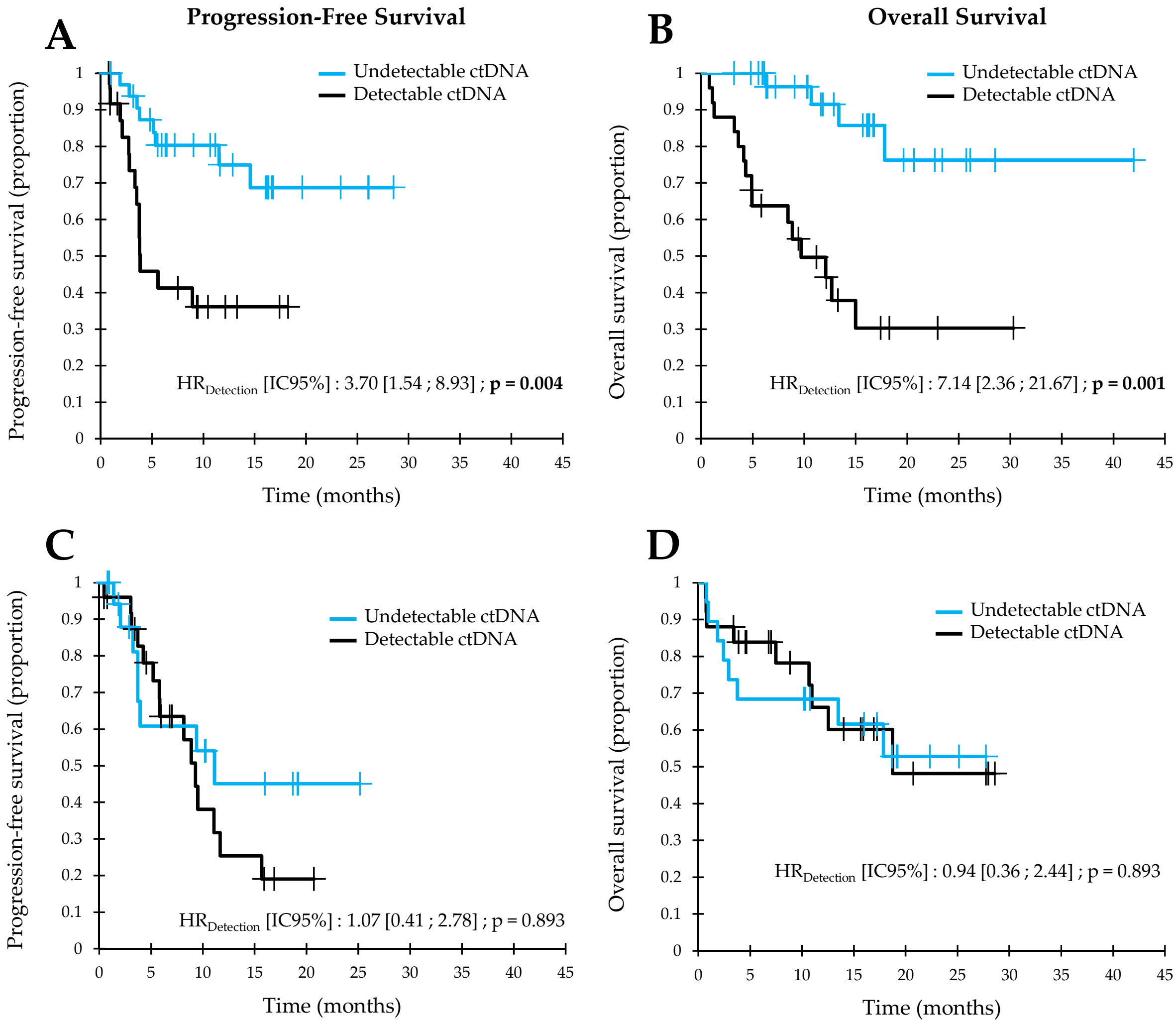

Figure S1. Prognostic value of baseline ctDNA detection in pooled analysis : Kaplan-Meier estimate of the PFS **(A)** and OS **(B)** depending on ctDNA detection at the initiation of first-line immunotherapy; Kaplan-Meier estimate of PFS **(C)** and OS **(D)** depending on ctDNA detection at the initiation of an immunotherapy beyond the first line.

Subgroup	n	HR	95% CI
**Overall**	57	**7.143**	**[2.355;21.669]**
**Sex**			
Male	22	**4.848**	**[1.037;47.713]**
Female	36	**6.685**	**[1.888;28.179]**
**Age at sampling**			
> 68 years	29	**6.833**	**[1.955;35.753]**
≤ 68 years	29	**6.042**	**[1.201;59.082]**
**Stage at sampling**			
Stage III	24	3.368	[0.545;18.042]
Stage IV	34	**9.079**	**[2.211;83.230]**
**Mutated gene**			
NRAS	51	**5.614**	**[2.082;18.475]**
BRAF	7	6.000	[0.320;875.521]
**Treatment**			
Nivolumab monotherapy	51	**9.540**	**[3.217;37.528]**
Nivolumab + Ipilimumab	7	0.471	[0.037;6.055]
**Thickness**			
> 2.3 mm	22	**7.056**	**[1.390;69.374]**
≤ 2.3 mm	23	**4.880**	**[1.356;20.855]**
**Ulceration**			
Yes	21	**6.567**	**[1.369;63.331]**
No	21	**22.361**	**[2.520;2940.303]**
**Baseline LDH (IU/L)**			
> 426	9	Not evaluable	
≤ 426	29	**6.725**	**[1.778;36.246]**
**Number of metastases**			
> 2	23	**15.234**	**[1.973;1958.451]**
≤ 2	35	3.711	[0.949;14.533]
**Presence of lymph node metastasis**			
Yes	41	**5.351**	**[1.621;27.258]**
No	17	**29.236**	**[2.335;4054.253]**
**Presence of cutaneous metastasis**			
Yes	33	**8.403**	**[2.554;35.312]**
No	25	**14.905**	**[1.687;1957.802]**
**Presence of pulmonary metastasis**			
Yes	14	**24.184**	**[2.925;3151.894]**
No	44	2.944	[0.887;10.695]
**Presence of abdominal metastasis**			
Yes	15	0.788	[0.144;7.908]
No	43	**10.013**	**[3.115;40.711]**
**Presence of cerebral metastasis**			
Yes	6	5.000	[0.407;689.614]
No	52	**6.168**	**[2.239;20.584]**
**Presence of bone metastasis**			
Yes	15	3.135	[0.379;407.628]
No	43	**4.094**	**[1.149;15.400]**
**Multivariate analysis** Adjustment on stage, treatment, mutated gene, LDH activity, number of metastasis, presence of lymph node, cutaneous, pulmonary, abdominal, cerebral or bone metastasis	57	**10.520**	**[1.828;60.545]**

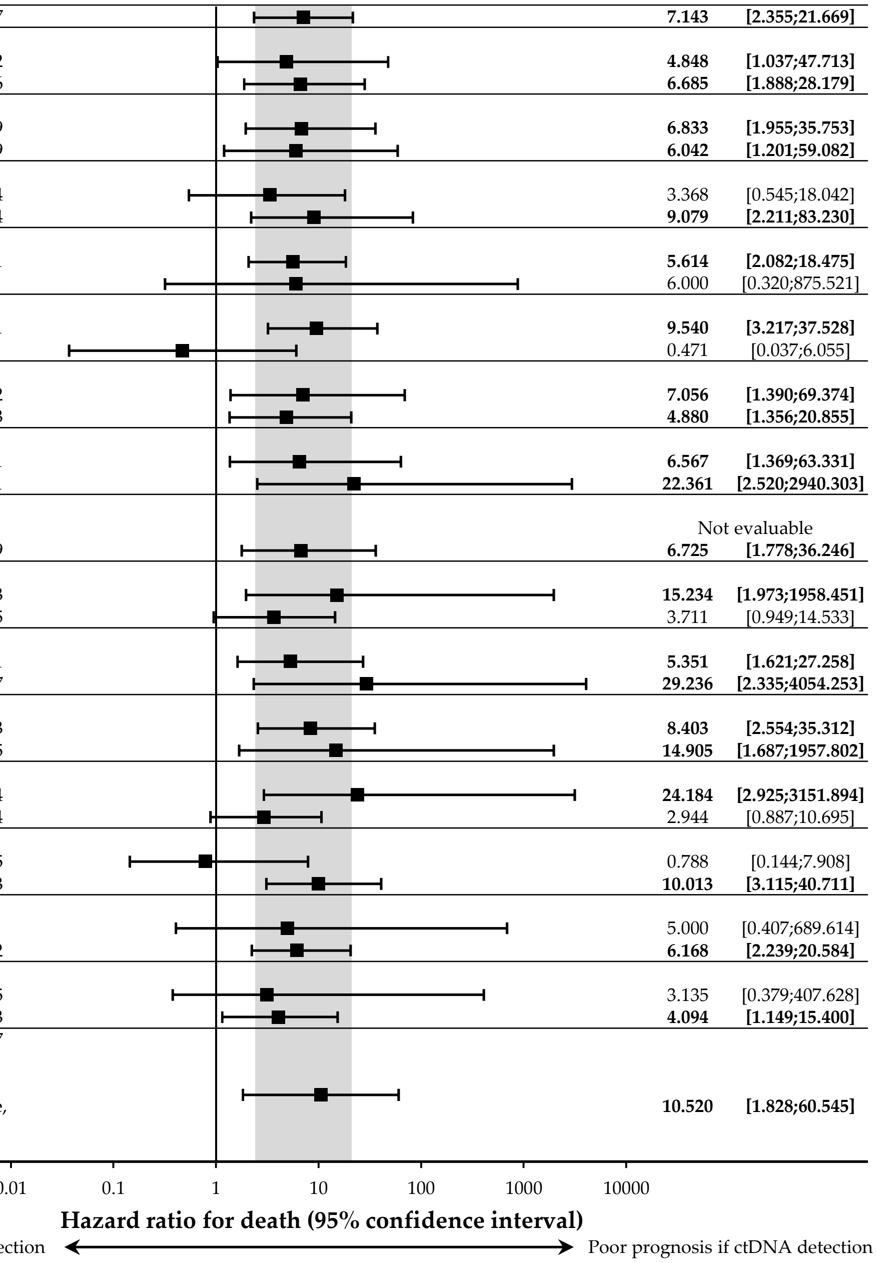

Figure S2. Forest plot of OS in subgroup and multivariate analysis, according to ctDNA detectability at the initiation of first-line immunotherapy.

Subgroup	n	HR	95% CI
**Overall**	57	**3.704**	**[1.537;8.926]**
**Sex**			
Male	22	1.604	[0.555;5.462]
Female	36	3.894	[0.985;15.547]
**Age at sampling**			
> 68 years	29	**4.628**	**[1.423;18.795]**
≤ 68 years	29	2.603	[0.812;2.618]
**Stage at sampling**			
Stage III	24	1.239	[0.231;4.661]
Stage IV	34	**10.700**	**[2.565;98.635]**
**Mutated gene**			
NRAS	51	**3.235**	**[1.372;8.089]**
BRAF	7	6.000	[0.320;875.521]
**Treatment**			
Nivolumab monotherapy	51	**4.120**	**[1.709;10.724]**
Nivolumab + Ipilimumab	7	1.225	[0.098;15.300]
**Thickness**			
> 2.3 mm	22	1.471	[0.391;5.243]
≤ 2.3 mm	23	**8.184**	**[2.004;45.943]**
**Ulceration**			
Yes	21	**4.487**	**[1.042;25.855]**
No	21	3.202	[0.898;12.024]
**Baseline LDH (IU/L)**			
> 426	9	Not evaluable	
≤ 426	29	**3.759**	**[1.191;13.339]**
**Number of metastases**			
> 2	23	**3.480**	**[1.010;18.086]**
≤ 2	35	2.210	[0.520;7.936]
**Presence of lymph node metastasis**			
Yes	41	2.389	[0.951;6.607]
No	17	**9.482**	**[1.253;104.089]**
**Presence of cutaneous metastasis**			
Yes	33	**3.184**	**[1.040;10.465]**
No	25	**5.258**	**[1.394;28.287]**
**Presence of pulmonary metastasis**			
Yes	14	4.716	[0.996;45.593]
No	44	2.534	[0.896;7.172]
**Presence of abdominal metastasis**			
Yes	15	4.622	[0.540;604.471]
No	43	**3.606**	**[1.344;9.716]**
**Presence of cerebral metastasis**			
Yes	6	3.000	[0.160;437.761]
No	52	**3.4698**	**[1.481;8.603]**
**Presence of bone metastasis**			
Yes	15	5.000	[0.611;649.39]
No	43	2.321	[0.737;6.727]
**Multivariate analysis** Adjustment on age, gender, stage, mutated gene, treatment, LDH, thickness, ulceration, number of metastasis, presence of lymph node, cutaneous, pulmonary, abdominal, cerebral or bone metastasis	57	**5.517**	**[1.223;24.899]**

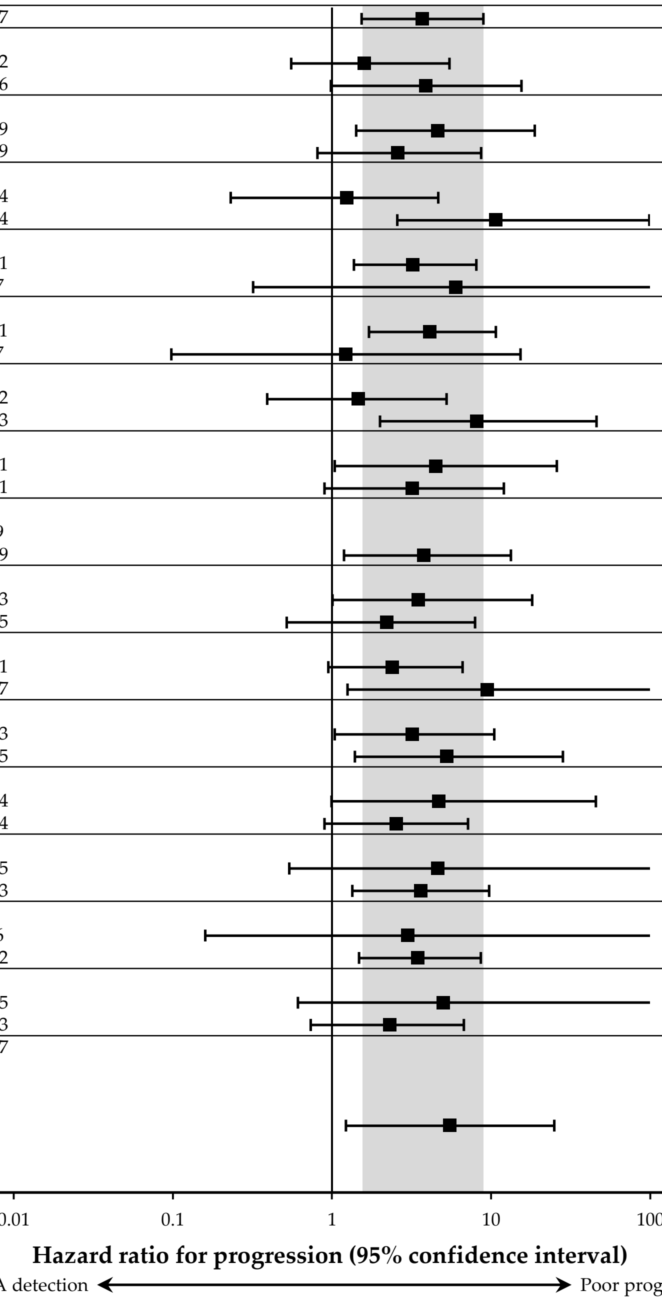

Figure S3. Forest plot of PFS in subgroup and multivariate analysis, according to ctDNA detectability at the initiation of first-line immunotherapy.
